# Exploring the association between cancer and cognitive impairment in the Australian Imaging Biomarkers and Lifestyle (AIBL) study

**DOI:** 10.1038/s41598-024-54875-3

**Published:** 2024-02-22

**Authors:** Liwei Ma, Yi Ling Clare Low, Yuanhao Zhuo, Chenyin Chu, Yihan Wang, Christopher J. Fowler, Edwin C. K. Tan, Colin L. Masters, Liang Jin, Yijun Pan

**Affiliations:** 1 Florey Institute of Neuroscience and Mental Health, The University of Melbourne, Parkville, VIC 3010 Australia; 2 The University of Sydney School of Pharmacy, Faculty of Medicine and Health, The University of Sydney, Sydney, NSW 2006 Australia; 3 Drug Delivery, Disposition and Dynamics, Monash Institute of Pharmaceutical Sciences, Monash University, Parkville, VIC 3052 Australia; 4 Department of Organ Anatomy, Graduate School of Medicine, Tohoku University, Sendai, Miyagi 980-8575 Japan

**Keywords:** Alzheimer’s disease, APOE, Cancer, Cohort study, Comorbidities, Mild cognitive impairment, Cognitive decline, Cancer, Epidemiology, Dementia, Alzheimer's disease

## Abstract

An inverse association between cancer and Alzheimer’s disease (AD) has been demonstrated; however, the association between cancer and mild cognitive impairment (MCI), and the association between cancer and cognitive decline are yet to be clarified. The AIBL dataset was used to address these knowledge gaps. The crude and adjusted odds ratios for MCI/AD and cognitive decline were compared between participants with/without cancer (referred to as C+ and C− participants). A 37% reduction in odds for AD was observed in C+ participants compared to C− participants after adjusting for all confounders. The overall risk for MCI and AD in C+ participants was reduced by 27% and 31%, respectively. The odds of cognitive decline from MCI to AD was reduced by 59% in C+ participants after adjusting for all confounders. The risk of cognitive decline from MCI to AD was halved in C+ participants. The estimated mean change in Clinical Dementia Rating-Sum of boxes (CDR-SOB) score per year was 0.23 units/year higher in C− participants than in C+ participants. Overall, an inverse association between cancer and MCI/AD was observed in AIBL, which is in line with previous reports. Importantly, an inverse association between cancer and cognitive decline has also been identified.

## Introduction

The presence of multiple chronic health conditions (referred to as comorbidities), such as cardiovascular disease, cancer, Alzheimer’s disease (AD), and diabetes, becomes more common as people age^1^. While the association between comorbidities and AD has been well studied, that between cancer and AD has not yet been comprehensively investigated. Most epidemiological studies have focused on cancer risk after the diagnosis of AD (exposure = AD, outcome = cancer) or AD risk in patients with cancer (exposure = cancer, outcome = AD). A reduction in cancer incidence rate was observed in individuals with AD, with the risk ratio (RR) ranging from 0.29 to 0.90^2–11^, and reduced incidence rate of AD was noted in people with cancer^2,3,6,10–19^. Although contradictory results are sometimes observed^14^, most studies suggest that cancer reduces the incidence rate of AD and vice versa.

There are knowledge gaps that remain to be filled to clarify the association between cancer and AD. First, mild cognitive impairment (MCI), an important transitional phase prior to the development of AD is usually neglected^2,10,11^ in epidemiological studies, and the association between cancer and MCI is still inconclusive^20^. Second, the association between cancer and cognitive decline in AD is unclear. Unravelling this epidemiological association will lend support to further studies exploring the underlying mechanisms contributing to the interaction between cancer and MCI/AD, which has the potential to impact on the development of novel strategies to intervene cognitive decline in AD. Last but not least, although the association between cancer and AD has been explored in European^6,13,16–18^ and American studies^2,3,10–12,14,15,19^, and the influence of geographic and demographic factors on this association was noted, an Australian study is lacking.

To address these knowledge gaps, we leveraged longitudinal data from the Australian Imaging Biomarkers and Lifestyle (AIBL) study^21,22^ and examined the risk for MCI/AD in participants with cancer and the risk for cognitive decline (i.e. cognitive unimpaired [CU] to MCI, CU to AD, and MCI to AD) due to cancer. Confounding factors were adjusted for or stratified. In addition, change in Clinical Dementia Rating Scale Sum of Boxes scores over time (ΔCDR-SOB, a surrogate for cognitive decline) was calculated and compared between participants with and without cancer.

## Material and methods

### Participants

**Figure 1:**
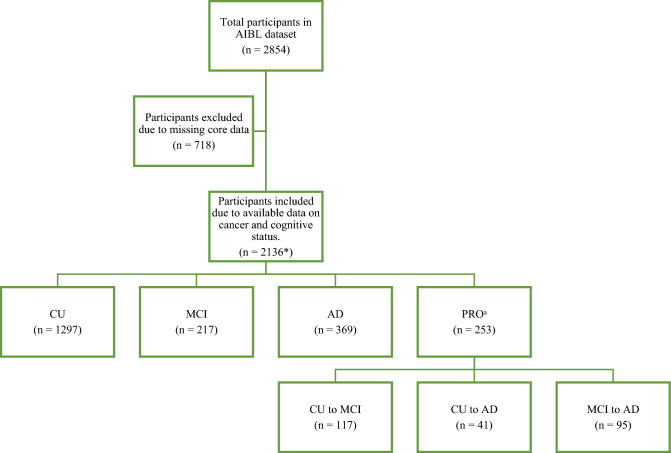
Study participants. The AIBL dataset included 2854 participants, of which 718 individuals were removed due to unknown cancer or cognitive status. 2136 participants remained for data analysis in the present study, with 1297 CU, 217 MCI and 369 AD participants. In addition, 253 participants exhibited changes in cognition category (i.e. cognitive decline) during follow-up period. Of these, 117 progressed from CU to MCI, 41 progressed from CU to AD, and 95 from MCI to AD. *CDR-SOB score was available for 1279 participants; APOE genotype was recorded for 1958 participants; smoking status, alcohol consumption, and years of education were known for 1934, 1830 and 2117 participants, respectively. *CU* cognitive unimpaired, *MCI* mild cognitive impairment, *AD* Alzheimer’s disease, *PRO* progression in cognition category during the follow-up period.

The AIBL study was approved by the institutional ethics committees at St. Vincent’s Health and the University of Melbourne, and all participants have provided written informed consent prior to study enrolment. All methods were performed in accordance with the Australian code for the responsible conduct of research. To date, 2854 participants have been recruited to AIBL. Longitudinal data was collected over an average 7.2-year period, with an overall participation rate of 84.9%. The selection process for participants in the current study is summarized in Fig. 1. Of the total 2854 AIBL participants, 718 participants were excluded from the study due to unknown cancer and/or cognitive status at enrolment. Of the included participants (n = 2136), 55.6% were female (n = 1187) and 44.4% were male (n = 949). The participants were categorized as CU, MCI, AD, and PRO (those who experienced progression in cognition category during the follow-up period). Cognitive assessment is detailed in the Clinical Diagnosis section below.

### History of cancer (exposure)

History of cancer was self-reported at enrolment and the occurrence of cancer was self-reported during the follow-up period. Cancer status of the participants was categorized as (1) self-report of cancer at enrollment or during follow-up (C+); (2) no self-report of cancer at enrolment or during follow-up (C−). For participants with change in cognition category, only those who were diagnosed with cancer before cognition category change were included in the analysis.

### Clinical diagnosis of MCI and AD (outcome)

The criteria used for clinical diagnosis of MCI and AD has been described in previous studies^21,22^. Cognitive status of the participants was determined by neurologists and neuropsychologists using amyloid PET imaging and neuropsychology tests. Participants who were identified as CU or MCI at enrolment but experienced cognition category change over the follow-up period (i.e. CU to MCI, CU to AD, and MCI to AD) were assigned to the PRO group. Participants who were identified as CU, MCI, or AD at enrolment and throughout the study were categorized into the CU, MCI, and AD groups.

### Potential confounders

Sex, APOEε4 (carrier or noncarrier), smoking (never, former, current), alcohol (non/light/moderate/heavy drinker), and education were determined from self-report and medical records. In addition, a blood sample was taken for APOE genetic testing for participants who gave consent. Light, moderate, and heavy alcohol consumption was defined as < 3 days per week, 3 to 6 days per week, and daily consumption, respectively. Descriptive statistics for the potential confounders are provided in Supplementary Data Table S1.

### Statistical analysis

Data analysis was performed using Stata statistical software (version 17.0; College Station, TX), where univariable/multivariable logistic regression and univariable linear regression programs were used. This study assessed (1) the odds ratio/risk ratio (OR/RR) for MCI and AD in C+ participants compared with C− participants; (2) the OR/RR of cancer exposure (C+) on cognitive decline (change of cognition category); (3) the impact of potential confounders on the associations measured in (1) and (2). In addition, the change in CDR-SOB score over time (ΔCDR-SOB) was compared between C+ and C− participants. As the time between the first and last measurement of the CDR-SOB score varied for each participant, the ΔCDR-SOB was normalized by time. All statistical tests performed were two-sided. Statistical significance and 95% confidence interval (CI) were determined, with statistical significance defined as p-value < 0.05. Figures were prepared by GraphPad Prism Software (Version 9.1.0) (Boston, MA).

## Results

### Characteristics of the study population

**Figure 2:**
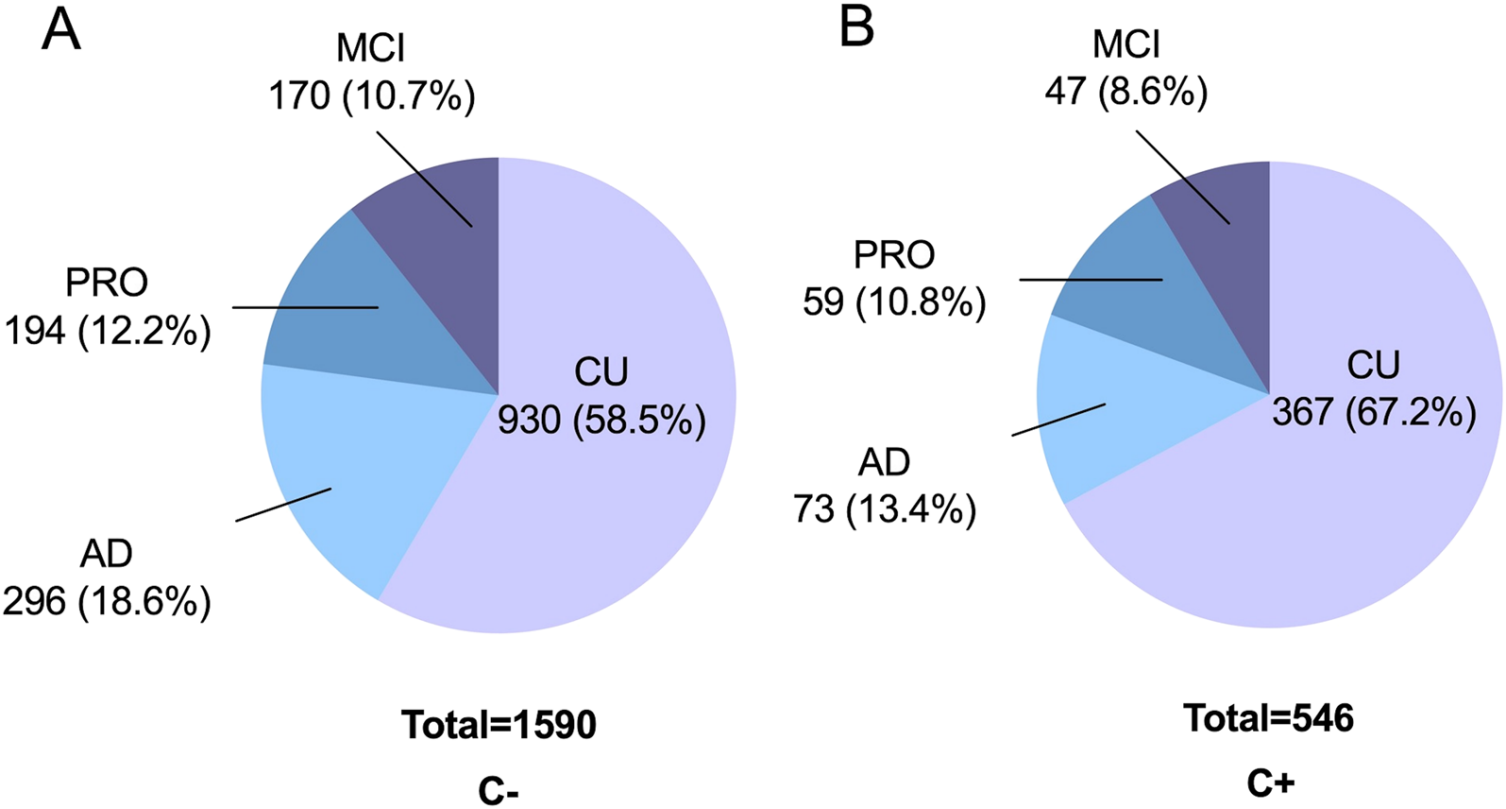
The proportions of each cognition category for AIBL participants with and without cancer (C+ and C−). (**A**) Of the 1590 C− participants, 58.5%, 10.7%, 18.6% and 12.2% were categorized as CU, MCI, AD and PRO, respectively. (**B**) Of the 546 C+ participants, 67.2%, 8.6%, 13.4%, and 10.8% were categorized as CU, MCI, AD and PRO, respectively. *CU* cognitive unimpaired, *MCI* mild cognitive impairment, *AD* Alzheimer’s disease, *PRO* progression in cognition category during the follow-up period.

A total of 2136 participants were included in the present study, including 1297 (60.7%) CU, 217 (10.2%) MCI, 369 (17.3%) AD and 253 (11.8%) PRO (Table 1). Within the PRO group, 117 (46.3%) participants progressed from CU to MCI, 41 (16.2%) participants progressed from CU to AD, and 95 (37.5%) participants progressed from MCI to AD. Of the 2136 participants, 1590 (74.4%) had never diagnosed with cancer (C−), and 546 (25.6%) had a history of cancer (C+) at the time of enrollment (Table 1). Of the 1590 C− participants, 930 (58.5%), 170 (10.7%), 296 (18.6%), 194 (12.2%) belonged to the CU, MCI, AD, and PRO groups, respectively (Fig. 2A). Of the 546 C + participants, 367 (67.2%), 47 (8.6%), 73 (13.4%) and 59 (10.8%) belonged to the CU, MCI, AD, and PRO groups, respectively (Fig. 2B). The averaged age and distribution of sex for participants under each cognition group is also provided in Table 1.

**Table 1:** Characteristics of the study participants.

	C+	C−	Total population	
CU (%)	367 (28.3)	930 (71.7)	1297	
MCI (%)	47 (21.7)	170 (78.3)	217	
AD (%)	73 (19.8)	296 (80.2)	369	
PRO (%)	59 (23.3)	194 (76.7)	253	
Total (%)	546 (25.6)	1590 (74.4)	2136	

	All cognitive status	CU	MCI	AD
Age, mean (SD), years				
C+	73.0 (7.3)	71.4 (6.5)	74.4 (7.2)	78.1 (8.4)
C−	72.1 (7.5)	70.5 (6.5)	73.7 (8.2)	75.2 (8.8)
Total	72.3 (7.5)	70.7 (6.5)	73.9 (8.0)	75.8 (8.8)
Sex, female (%)				
C+	261 (47.8)	184 (50.1)	22 (46.8)	33 (45.2)
C−	926 (58.2)	573 (61.6)	86 (50.6)	170 (57.4)
Total	1187 (55.6)	757 (58.4)	108 (50.0)	203 (55.0)

Upper panel: the percentage of C+ and C− participants under each cognition category is shown in brackets. Lower panel: For age, the standard deviation of the age of the C+ and C− participants in each cognition category is shown in brackets. For sex, the percentage of female C+ and C− participants in each cognition category is shown in brackets.

### The association (OR) between cancer and AD is significant but not for cancer and MCI

To measure the association between cancer and MCI/AD, the crude and adjusted ORs were calculated (Table 2). Of the 217 MCI participants, 47 (21.7%) had cancer. A crude logistic regression model revealed a 0.70-fold relative decrease (OR 0.70 [0.50–0.99], p = 0.043) in the odds of MCI in C+ participants when compared to C− participants. The association between cancer and MCI remained to be statistically significant after individual adjustment for sex (0.67 [0.48–0.95], p = 0.025), APOE ε4 (0.65 [0.43–0.99], p = 0.046), and smoking (0.68 [0.48–0.98], p = 0.019). However, the association became statistically nonsignificant after adjusting for all confounders (0.62 [0.38–1.01], p = 0.056) (Table 2, + *All*, column 1). In summary, there was no significant inverse association between cancer and MCI after all confounders were adjusted for. Of the 369 AD participants, 73 (19.7%) had cancer. Crude regression model revealed that cancer was associated with approximately 40% lower odds of AD (0.62 [0.47–0.83], p = 0.001). Similar observations were also noted in individual confounder-adjusted regression models (Table 2, + *Sex*, + *APOE ε4*, + *Smoking*, + *Alcohol*, + *Education*, column 2). The odds of AD were significantly reduced by 0.63-fold (0.63 [0.43–0.92], p = 0.017) in C + participants compared to C- participants after adjusting for all confounders (Table 2, + *All*, column 2). In conclusion, an inverse association between cancer and AD was observed and remained significant even after adjusting for all confounders in this study.

**Table 2:** Odds of MCI & AD in participants with cancer.

	Odds of MCI in C+ participants	Odds of AD in C+ participants
Unadjusted	0.70 [0.50–0.99], p = 0.043	0.62 [0.47–0.83], p = 0.001
*+ Sex*	0.67 [0.48–0.95], p = 0.025	0.61 [0.46–0.81], p = 0.001
*+ APOE ε4*	0.65 [0.43–0.99], p = 0.046	0.67 [0.48–0.92], p = 0.013
*+ Smoking*	0.68 [0.48–0.98], p = 0.019	0.58 [0.44–0.77], p < 0.001
*+ Alcohol*	0.71 [0.49–1.02], p = 0.066	0.61 [0.45–0.81], p = 0.001
*+ Education*	0.76 [0.53–1.01], p = 0.127	0.67 [0.50–0.90], p = 0.008
*+ All*	0.62 [0.38–1.01], p = 0.056	0.63 [0.43–0.92], p = 0.017

Numbers in the brackets represent 95% CI.

### The association (RR) between cancer and MCI/AD is moderated by sex and APOE ε4 allele type

The crude risk of MCI (RR 0.73 [0.54–0.99], p = 0.042) and AD (RR 0.69 [0.54–0.87], p = 0.001) was significantly reduced in C+ participants when compared to C− participants (Table 3). Stratum-specific RR for confounders (sex and APOE ε4-allele type) was calculated. The risk reduction for the occurrence of MCI (0.63 [0.42–0.96], p = 0.025) or AD (0.69 [0.50–0.95], p = 0.018) remained statistically significant in male C+ participants compared to male C− participants. However, only the risk reduction of AD (RR 0.66 [0.47–0.93], p = 0.015), but not that of MCI, in female C + participants compared to female C- participants was statistically significant (Table 3, + *Sex*, columns 1 & 2). When examining the stratum-specific RR for the APOE ε4 allele type, only the risk reduction for the occurrence of AD (0.74 [0.55–0.99], p = 0.036), but not that for MCI, reached statistical significance for C + participants carrying the APOE ε4 allele compared with C- participants (Table 3, + *APOE ε4*, column 2). When the data were stratified by both sex and APOE ε4 allele type, all RRs were not statistically significant, except for male APOE ε4 carriers (RR 0.40 [0.16–0.99], p = 0.031). Detailed statistics can be found in Supplementary Data Table S2. In conclusion, a significant inverse association was observed between cancer and MCI/AD. However, the stratum-specific RR showed that the association between cancer and MCI/AD contained differences for each stratum of the confounder. The stratum-specific RR was not calculated for smoking (three subgroups), alcohol (four subgroups), education (six subgroups), or their combinations, given the small sample size resulting from stratification.

**Table 3:** Risk of MCI & AD in participants with cancer.

	Risk of MCI in C+ participants	Risk of AD in C+ participants
Crude	0.73 [0.54–0.99], p = 0.042	0.69 [0.54–0.87], p = 0.001
+ *Sex*		
Male	0.63 [0.42–0.96], p = 0.025	0.69 [0.50–0.95], p = 0.018
Female	0.82 [0.53–1.27], p = 0.369	0.66 [0.47–0.93], p = 0.015
+ *APOE ε4*		
Carrier	0.59 [0.33–1.06], p = 0.066	0.74 [0.55–0.99], p = 0.036
Non-carrier	0.76 [0.46–1.27], p = 0.289	0.74 [0.48–1.14], p = 0.162

Numbers in the brackets represent 95% CI.

### The association (RR) between cancer and cognitive decline is moderated by different PRO categories

The association between cancer and cognitive decline was examined in the PRO group (Table 4, *column 1*), and no significant association was observed from either crude or adjusted models. Different categories of PRO (i.e. CU to MCI, MCI to AD, and CU to AD) were therefore analyzed meticulously. Progression from CU to MCI occurred in 88 participants, of whom 29 (24.79%) were C+. Unadjusted and adjusted logistic regression models revealed a nonsignificant association between cancer and progression from CU to MCI (Table 4, *column 2*). A total of 41 participants experienced cognitive decline from CU to AD, of whom 15 (36.59%) were C+; however, no statistically significant association was observed (Table 4, *column 3*). The same analyses were performed for participants who progressed from MCI to AD (n = 95), of whom 15 (15.79%) were C+. The crude logistic regression model showed a 0.48-fold relative decrease (OR 0.48 [0.27–0.84], p = 0.010) in the progression from MCI to AD in C+ participants compared with C− participants. In adjusted logistic regression models, cancer was inversely associated with the progression from MCI to AD, after separate adjustment for each confounder (ORs 0.45–0.51). A similar association was also found when all confounders were accounted for (0.41 [0.21–0.82], p = 0.011), and all these odds ratios reached statistical significance (Table 4, *column 4*).

**Table 4:** The association (OR) between cancer and cognitive decline.

	PRO	CU to MCI	CU to AD	MCI to AD
Unadjusted	0.77 [0.56–1.06], p = 0.106	0.84 [0.54–1.29], p = 0.419	1.46 [0.77–2.79], p = 0.250	0.48 [0.27–0.84], p = 0.010
+ *Sex*	0.73 [0.53–1.00], p = 0.051	0.80 [0.52–1.24], p = 0.317	1.35 [0.70–2.59], P = 0.372	0.45 [0.25–0.79], p = 0.006
+ *APOE ε4*	0.79 [0.57–1.09], p = 0.145	0.84 [0.53–1.30], p = 0.434	1.48 [0.77–2.84], p = 0.241	0.50 [0.28–0.89], p = 0.018
+ *Smoking*	0.77 [0.57–1.09], p = 0.145	0.86 [0.55–1.34], p = 0.494	1.37 [0.71–2.64], p = 0.349	0.50 [0.28–0.88], p = 0.017
+ *Alcohol*	0.80 [0.57–1.11], p = 0.184	0.89 [0.57–1.40], p = 0.623	1.38 [0.67–2.75], p = 0.365	0.46 [0.24–0.86], p = 0.016
+ *Education*	0.83 [0.60–1.14], p = 0.251	0.89 [0.57–1.39], p = 0.617	1.50 [0.78–2.87], p = 0.225	0.51 [0.29–0.91], p = 0.021
+ *All*	0.78 [0.55–1.13], p = 0.189	0.93 [0.59–1.49], p = 0.776	1.07 [0.51–2.23], p = 0.855	0.41 [0.21–0.82], p = 0.011

Numbers in the brackets represent 95% CI.

### The association (RR) between cancer and progression from MCI to AD is significant, while that between cancer and progression from CU to MCI or CU to AD is nonsignificant

The risk reduction of cognitive decline in C+ compared to C− participants was not statistically significant (0.80 [0.61–1.05], p = 0.105) (Table 5, *PRO*). RRs in each cognitive decline category were therefore assessed. Only the association between cancer and progression from MCI to AD was significant but not for any other cognitive decline categories. A 0.5-fold reduction (0.50 [0.29–0.85], p = 0.008) in the risk of progression from MCI to AD was observed in C+ participants compared with C− participants (Table 5, *MCI to AD*). The stratum-specific RR was not calculated for sex (two subgroups), APOE ε4 allele type (two subgroups), smoking (three subgroups), alcohol (four subgroups), education (six subgroups), or their combinations due to small sample size resulting from subgrouping that were impractical for analysis.

**Table 5:** The association (RR) between cancer and cognitive decline.

	RR [95% CI], p-value
PRO	0.80 [0.61–1.05], p = 0.105
CU to MCI	0.85 [0.57–1.27], p = 0.418
CU to AD	1.44 [0.77–2.70], p = 0.247
MCI to AD	0.50 [0.29–0.85], p = 0.008

### The increase in CDR-SOB score over time is slower in participants with history of cancer than those without

**Figure 3:**
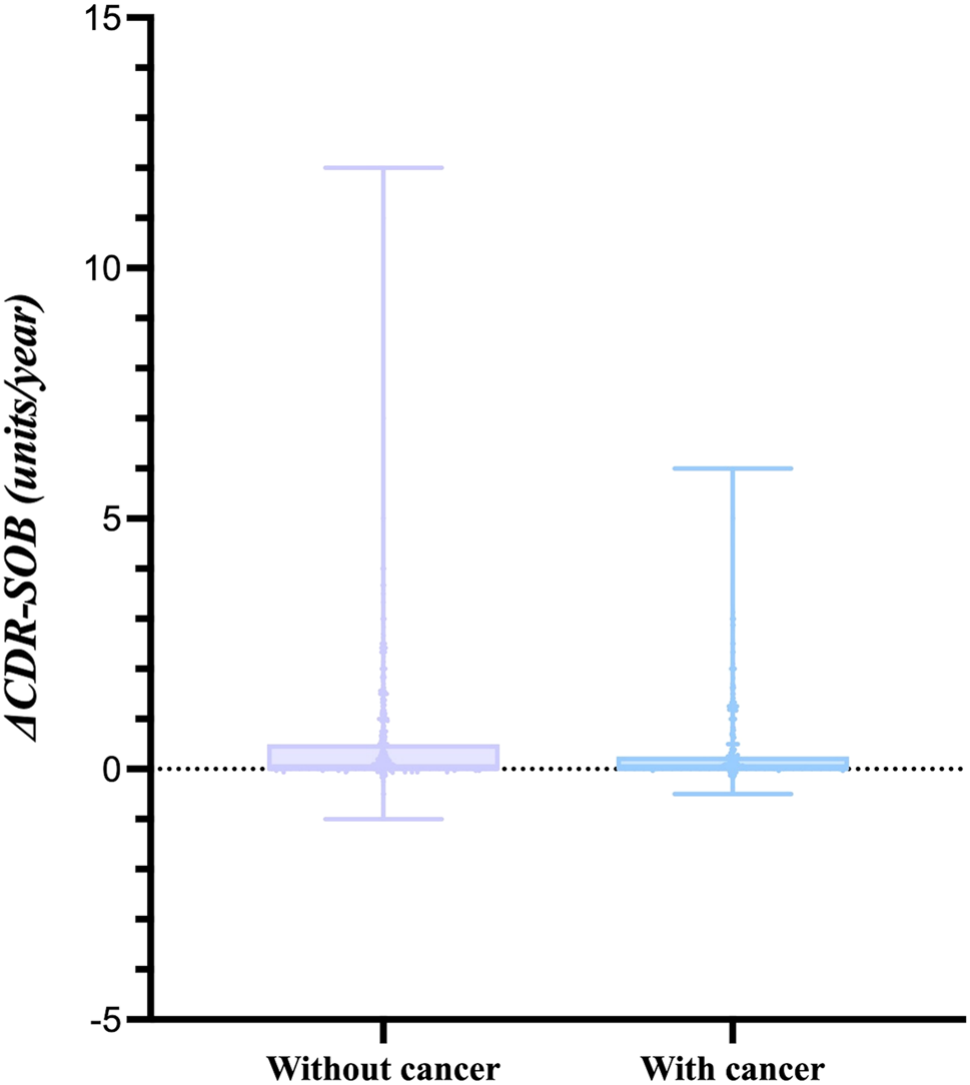
ΔCDR-SOB for AIBL participants with/without cancer*.* The ΔCDR-SOB was plotted for C− (without cancer, left boxplot) and C+ (with cancer, right boxplot) participants. The lowest ΔCDR-SOB for C− participants was −1 and the highest was 12. The lowest ΔCDR-SOB for C+ participants were −0.5 and the highest was 6. The mean ΔCDR-SOB for C− and C+ was 0.57 and 0.34 units/year, respectively.

The CDR-SOB is a global score regularly used in clinical and research settings to stage dementia severity^23^. Generally, more severe cognitive impairment was associated with a higher score. To understand the association between cancer and cognitive decline, the estimated mean change in CDR-SOB scores normalized by time (ΔCDR-SOB) was compared between C+ and C− participants (Fig. 3). The ΔCDR-SOB score was 0.34 and 0.57 units/year for C+ and C− participants, respectively (Table 6).

**Table 6:** The association between the CDR-SOB score over time and cancer history status.

	Coefficient	95% CI	p-value
ΔCDR-SOB	−0.23	[−0.38 to −0.08]	0.003

Mean change for ΔCDR-SOB between C+ and C− participants.

## Discussion

The present study examined the association between cancer and MCI/AD in AIBL participants. We noted that C+ participants were less likely to develop AD than C− participants, which is consistent with previous studies^6,11,16–18,24,25^. Our studies have also revealed a nonsignificant association between cancer and MCI. It must be emphasized that studying the association between cancer and MCI is challenging as MCI can be caused by different conditions, including AD, Lewy body dementia, Parkinson’s disease and even chemotherapy^26–29^. In future studies, MCI should be carefully characterized to eliminate disease heterogeneity from analysis; for example, the brain levels of amyloid-beta (Aβ) can be assessed, so that the association between cancer and Aβ-positive MCI can be precisely investigated.

The strength of our study is that we employed a large longitudinal dataset from AIBL to study the association between cancer and cognitive decline, which has not yet been comprehensively studied. The cognition category for the participants was clearly defined in our longitudinal study, so that we can ensure the temporal sequence between exposure (i.e. cancer) and the outcome (i.e. change in cognition category) to avoid reverse causality. All participants were included in the study for data analysis, except for those who had no record for cancer history or cognitive status, which reduced the risk of survival bias. The average age and gender distribution of the current study was comparable to a community-based cohort study conducted by University of Kentucky Alzheimer’s Disease Research Center (UK-ADRC)^30^. We showed that C+ participants were less likely to progress from MCI to AD compared with C− participants, while no significant association between cancer and cognitive decline from CU to MCI was observed. This observation has demonstrated the potential role of cancer on the progression of MCI to AD, but not on the development of MCI. The association between cancer and cognitive decline from CU to AD (n = 41) was found to be nonsignificant, which is not consistent with the association between cancer and change in ΔCDR-SOB score over time. Comparing the estimated mean change in ΔCDR-SOB score per year, it is noted that cognitive decline is slower in C+ participants than C− participants (n = 546 for C+, n = 1590 for C−). This discrepancy could be due to a much smaller sample size available for the former analysis approach.

In our study, there are several limitations that need to be considered, which may restrict the inference of a true association between exposure and outcome. History of cancer was self-reported by participants, and therefore recall bias is possible, especially in old participants. In addition, details of chemotherapy and types of cancer were not recorded. Therefore, an investigation of the association between cancer therapies or specific cancer types and subsequent development of MCI, AD, or PRO was not possible. A systematic review has revealed that various types of cancer can have distinct effects on the risk of subsequent cognitive decline. It suggests that the association between cancer and disease progression is not uniform across all cancer types^24^. Therefore, recording more details on cancer subtypes should be considered for future AIBL studies. In addition, the composition of our cohort was mainly Caucasians residing in Victoria (60%), and Western Australia (40%). This will limit the generalizability of our results to Australians residing in regions outside of our study area and Australians with diverse ethnic backgrounds. In addition, the AIBL study excludes participants who had cancer in the past 5 years from recruitment (excluding skin or in situ prostate cancer), which can potentially contribute to selection bias.

**Figure 4:**
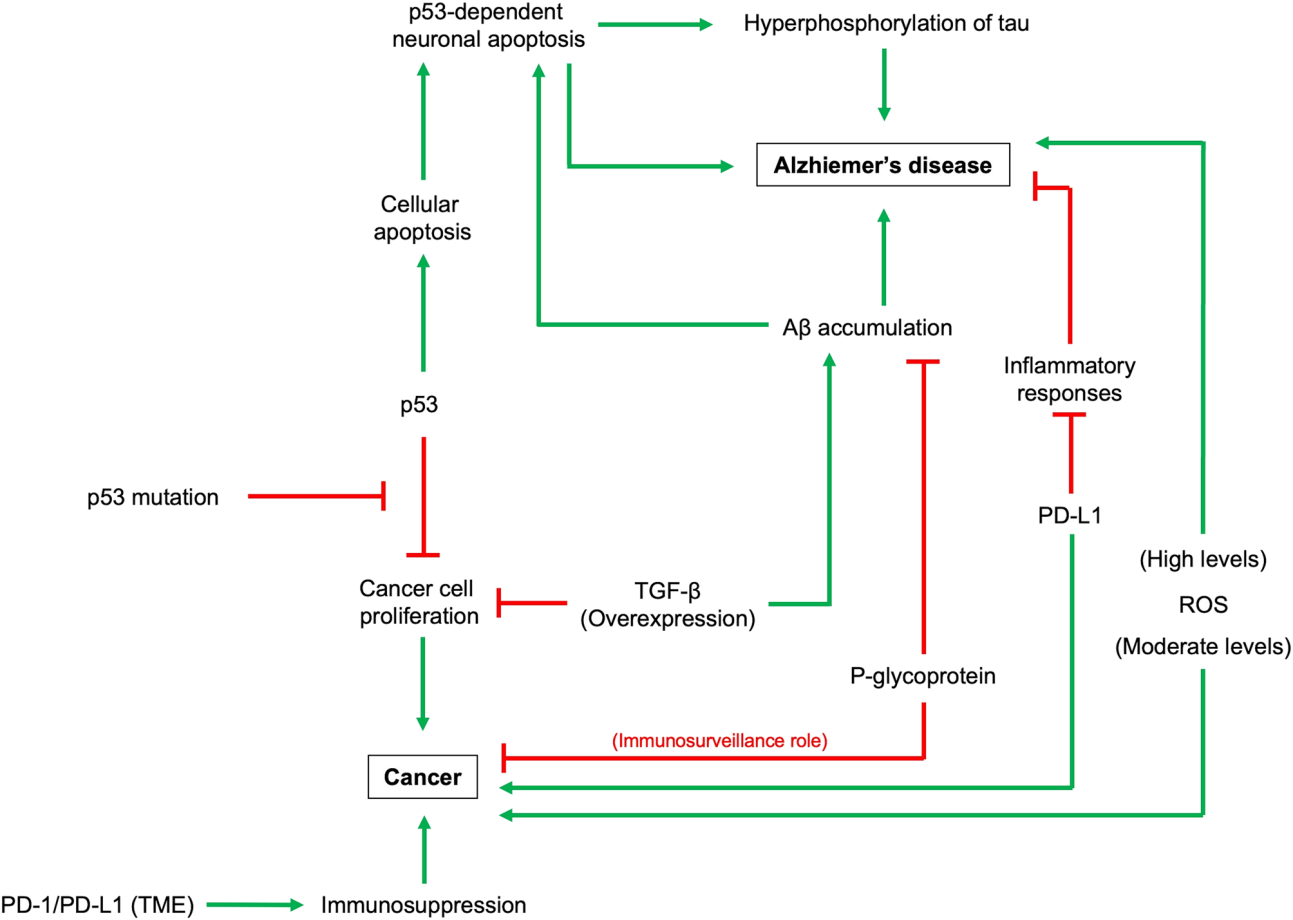
The proposed pathobiological interaction between cancer and Alzheimer’s disease. *PD-L1* programmed death-ligand 1, *PD-1*  programmed cell death protein 1, *ROS* reactive oxygen species, *TGF-β* transforming growth factor-β, *TME* tumor microenvironment; *green* promoting, *red* inhibiting.

The inverse association between cancer and AD can possibly be explained by the underlying pathobiological processes. At molecular level, the TP53 gene encodes the p53 protein, which is a well-recognized tumor suppressor^31,32^. In most cancer cases, the p53 gene is mutated and loses its tumor suppressive effect^33^.The accumulation of Aβ and hyperphosphorylation of tau are the hallmarks of AD^34^. Aβ promotes p53-dependent neuronal apoptosis by activating the p53 promoter, which is believed to be responsible for neurological disorders, such as AD^35,36^. The hyperphosphorylation of tau is also p53-dependent^37^. Other oncogenic molecules may also be involved in the inverse relationship between cancer and AD. Transforming growth factor beta (TGF-β) overexpression upregulates the expression of amyloid precursor protein (APP) and contribute to Aβ accumulation, while producing anti-proliferative effect that may suppress cancer^38,39^. Tumor overexpress PD-L1 helps tumor survival by suppressing the immune system^40,41^. Interestingly, PD-L1 suppresses neuroinflammation and the associated AD pathology^42^. The mitochondria regulate cell survival or apoptosis via regulating the production of reactive oxygen species (ROS). Moderate ROS production promotes the cancer cells growth and proliferation, while high levels of ROS in AD promote neurodegeneration^43^. P-glycoprotein at the blood–brain barrier clears Aβ from the brain^44^, while P-glycoprotein plays an immunosurveillance role in cancer^45^. A hypothetical pathobiological interaction between cancer and AD is illustrated in Fig. 4.

In conclusion, we observed a negative association between cancer and MCI/AD in AIBL, which is consistent with previous studies. We also highlighted a lower incidence and prevalence of MCI/AD progression in participants with cancer. In addition, cancer is associated with cognitive decline (change of CDR-SOB score) at a slower rate. Further studies are required to investigate the pathobiology underlying the two diseases to explain their inverse association observed in epidemiological studies.

### Supplementary Information

Supplementary Tables.

## Data Availability

The dataset generated during and/or analyzed during the current study are available from the corresponding author on reasonable request, and subject to the approval by AIBL scientific committee.
