# Supplementary material for: Exploring the association between cancer and cognitive impairment in the Australian Imaging Biomarkers and Lifestyle (AIBL) study
Source: Sci Rep. 2024 Feb 22;14:4364. doi: 10.1038/s41598-024-54875-3 (PMC10884016; doi:10.1038/s41598-024-54875-3)
Supplement: Supplementary file 1 — Supplementary Tables. [file 41598_2024_54875_MOESM1_ESM.docx]

**SUPPLEMENTARY DATA**

**Table S1: The descriptive statistics on cognition and cancer status of participants by potential confounders.**

| **Variable** | **All participants**  **(n = 2136)** | | | | | | | |
| --- | --- | --- | --- | --- | --- | --- | --- | --- |
|  | **Cognitive status** | | | |  | **History of cancer** | |  |
|  | **CU** | **MCI** | **AD** | **PRO** | ***p*-value** | **No** | **Yes** | ***p*-value** |
| ***Sex*** |  |  |  |  | 0.002 |  |  | <0.001 |
| *male* | 540  (57%) | 109  (12%) | 166  (17%) | 134  (14%) |  | 664  (70%) | 285  (30%) |  |
| *female* | 757  (64%) | 108  (9%) | 203  (12%) | 119  (10%) |  | 926  (78%) | 261  (22%) |  |
| ***APOE ε4 status*** |  |  |  |  | <0.001 |  |  | 0.122 |
| *carrier* | 328  (46%) | 70  (10%) | 190  (27%) | 127  (18%) |  | 545  (76%) | 170  (24%) |  |
| *non-carrier* | 931  (75%) | 79  (6%) | 109  (9%) | 124  (10%) |  | 908  (73%) | 335  (27%) |  |
| ***Smoking*** |  |  |  |  | <0.001 |  |  | <0.001 |
| *never* | 737  (63%) | 101  (9%) | 196  (17%) | 138  (12%) |  | 893  (76%) | 279  (24%) |  |
| *former* | 382  (54%) | 88  (12%) | 148  (21%) | 92  (13%) |  | 488  (69%) | 222  (31%) |  |
| *current* | 35  (67%) | 7  (13%) | 5  (10%) | 5  (10%) |  | 42  (81%) | 10  (19%) |  |
| ***Alcohol consumption*** |  |  |  |  | <0.001 |  |  | 0.486 |
| *none* | 157  (46%) | 34  (10%) | 104  (31%) | 46  (13%) |  | 252  (74%) | 89  (26%) |  |
| *light* | 215  (63%) | 44  (13%) | 53  (16%) | 27  (8%) |  | 252  (74%) | 87  (26%) |  |
| *moderate* | 506  (69%) | 50  (7%) | 80  (11%) | 99  (13%) |  | 545  (74%) | 190  (26%) |  |
| *alcoholics* | 219  (53%) | 59  (14%) | 90  (22%) | 47  (11%) |  | 301  (73%) | 114  (27%) |  |
| ***Education*** |  |  |  |  | <0.001 |  |  | <0.001 |
| *0-6* | 4  (13%) | 5  (16%) | 20  (65%) | 2  (6%) |  | 26  (84%) | 5  (16%) |  |
| *7-8* | 74  (43%) | 26  (15%) | 46  (27%) | 26  (15%) |  | 137  (80%) | 35  (20%) |  |
| *9-12* | 440  (55%) | 102  (13%) | 140  (18%) | 113  (14%) |  | 610  (77%) | 185  (23%) |  |
| *13-15* | 283  (64%) | 38  (9%) | 73  (17%) | 45  (10%) |  | 332  (76%) | 107  (24%) |  |
| *15+* | 493  (73%) | 44  (6%) | 78  (11%) | 65  (10%) |  | 468  (69%) | 212  (31%) |  |

CU = cognitive unimpaired, MCI = mild cognitive impairment, AD = Alzheimer’s disease, PRO = progression in cognition category during the follow-up period

**Table S2: Risk of MCI & AD in participants with cancer history.**

|  | **Risk of MCI in participants with cancer** | **Risk of AD in participants with cancer** |
| --- | --- | --- |
| **Female APOE ε4-** | RR 0.87 [0.42 – 1.78], *p* = 0.701 | RR 0.83 [0.47 – 1.46], *p* = 0.515 |
| **Female APOE ε4+** | RR 0.81 [0.38 – 1.72], *p* = 0.572 | RR 0.72 [0.46 – 1.13], *p* = 0.130 |
| **Male APOE ε4-** | RR 0.64 [0.31 – 1.31], *p* = 0.217 | RR 0.63 [0.32 – 1.24], *p* = 0.177 |
| **Male APOE ε4+** | RR 0.40 [0.16 – 0.99], *p* = 0.031 | RR 0.72 [0.48 – 1.08], *p* = 0.098 |

Combination of two potential confounders (sex and APOE status)
